# Acoustic resolution photoacoustic Doppler velocimetry in blood-mimicking fluids

**DOI:** 10.1038/srep20902

**Published:** 2016-02-19

**Authors:** Joanna Brunker, Paul Beard

**Affiliations:** 1 Department of Medical Physics and Biomedical Engineering, University College London, Gower Street, London WC1E 6BT, UK

## Abstract

Photoacoustic Doppler velocimetry provides a major opportunity to overcome limitations of existing blood flow measuring methods. By enabling measurements with high spatial resolution several millimetres deep in tissue, it could probe microvascular blood flow abnormalities characteristic of many different diseases. Although previous work has demonstrated feasibility in solid phantoms, measurements in blood have proved significantly more challenging. This difficulty is commonly attributed to the requirement that the absorber spatial distribution is heterogeneous relative to the minimum detectable acoustic wavelength. By undertaking a rigorous study using blood-mimicking fluid suspensions of 3 μm absorbing microspheres, it was discovered that the perceived heterogeneity is not only limited by the intrinsic detector bandwidth; in addition, bandlimiting due to spatial averaging within the detector field-of-view also reduces perceived heterogeneity and compromises velocity measurement accuracy. These detrimental effects were found to be mitigated by high-pass filtering to select photoacoustic signal components associated with high heterogeneity. Measurement under-reading due to limited light penetration into the flow vessel was also observed. Accurate average velocity measurements were recovered using “range-gating”, which furthermore maps the cross-sectional velocity profile. These insights may help pave the way to deep-tissue non-invasive mapping of microvascular blood flow using photoacoustic methods.

The measurement of blood flow velocity can aid the diagnosis and treatment of numerous clinical conditions, ranging from atherosclerosis^1^, diabetes^2^ and cancer^3^. Whilst many imaging modalities have been implemented to measure blood flow^4^, each method has limitations. Optical microscopy techniques^5^ enable high-resolution flow imaging but are limited to depths of approximately 1 mm beyond which the resolution is degraded by the strong scattering of light in tissue. Deep tissue non-invasive blood flow measurements can be achieved using Doppler ultrasound^6^. However, its utility is mainly limited to measurements of blood flow in large arteries and veins since smaller vessels generate less backscattered ultrasound and exhibit slower flow speeds that are difficult to distinguish from the movements of the surrounding tissue.

Photoacoustic flowmetry (PAF) and velocimetry techniques^7^ have the potential to overcome the limitations of existing methods for measuring blood flow. The general principle of photoacoustic flowmetry involves illuminating red blood cells with modulated laser light in order to generate ultrasound (photoacoustic) waves via the photoacoustic effect. The velocity of moving red blood cells can then be calculated from measureable changes such as time, phase or frequency shifts in the photoacoustic waves they emit. The difficulty in Doppler ultrasound of detecting flow in microvessels is alleviated using photoacoustic methods on account of the strong optical absorption contrast between blood vessels and neighbouring tissues. In addition, by encoding light absorption onto acoustic waves, there is the potential to avoid the limited penetration depth of optical methods, which rely on unscattered (ballistic) photons.

In order to exploit the potential for deep tissue photoacoustic flowmetry, it is necessary to employ diffuse illumination, rather than ballistic photons. Measurements in the diffusive illumination regime must therefore utilise acoustic rather than optical focussing in order to localise the photoacoustic signal: this is the so-called “acoustic resolution” mode of photoacoustic flowmetry (AR-PAF) with the acoustic focusing being achieved using a focused detector or equivalently synthesized with an array of receivers. However, photoacoustic flowmetry has so far only been demonstrated *in vivo* using the “optical resolution” mode (OR-PAF), where the light is tightly focussed to a diffraction limited spot size comparable to individual red blood cells, thus limiting the penetration depth to less than 1 mm^8^,^9^,^10^,^11^,^12^,^13^. In OR-PAF it is relatively straightforward to directly track individual red blood cells as they move through the optical beam since the focussed spot is of comparable dimensions to a single red blood cell. Achieving accurate AR-PAF measurements in tissue realistic fluid phantoms or blood appears to be significantly more challenging^14^,^15^. It has been suggested that this is because of the need for the blood to exhibit a certain level of heterogeneity relative to the intrinsic detection bandwidth^7^,^15^,^16^,^17^. In other words, if the spatial separation of the red blood cells in whole blood is significantly smaller than the minimum detectable acoustic wavelength, the blood, as perceived by the acoustic detector, will approximate to a spatial continuum that is indistinguishable from a stationary absorbing medium. Under these conditions it will not be possible to detect flow.

The purpose of this paper is to explore whether the above heterogeneity issue is indeed the limiting factor in AR-PAF and, if it is, whether it can be mitigated in order to make accurate measurements in whole blood. To achieve this, a time correlation based AR-PAF approach^18^ was employed in conjunction with a well controlled and repeatable fluid blood flow phantom comprising aqueous suspensions of 3 μm absorbing polystyrene microspheres. This time correlation AR-PAF approach has previously been shown to provide a high level of quantitative accuracy (<1%) when using a solid moving phantom comprising a distribution of micron-scale absorbing features printed on to a polymer sheet^19^. However when applied to flow measurements of absorbing particles suspended in a fluid (including blood)^14^,^15^, it proved very challenging to achieve accurate measurements.

This paper begins with a description of the time correlation technique that was used to acquire velocity measurements in the above-mentioned microsphere blood flow phantom. These measurements are then presented for various microsphere concentrations, and flowing in tubes of different diameters. The observed measurements are discussed in terms of the degree of absorber heterogeneity and its influence on the frequency content of the photoacoustic signal as well as the beneficial effect of filtering the signal on measurement accuracy. In addition, a second deleterious effect is observed relating to limited light penetration into the vessel, and this is addressed using a new signal processing method, analogous to “range-gating” in Doppler ultrasound, which provides improved measurement accuracy, as well as mapping of the spatial velocity profile. These findings offer the prospect of overcoming the challenges in making acoustic resolution photoacoustic flow measurements and paving the way to deep tissue non-invasive mapping of blood flow in the microvasculature.

## Acoustic resolution photoacoustic Doppler velocimetry

Velocity measurements were made in fluids using a time-correlation Doppler flowmetry approach, which is described in detail in reference 17. The basic principle is predicated on the assumption that the fluid is composed of a random distribution of absorbing particles, which give rise to a unique photoacoustic signature when illuminated with laser pulses. Two laser pulses separated by a time *T* will generate a pair of photoacoustic waveforms which are near-identical in shape but shifted in time due to the motion of the fluid. Cross-correlation of the two waveforms then allows calculation of the measured time shift and thus the absolute absorber velocity.

## Blood-mimicking phantoms

**Figure 1:**
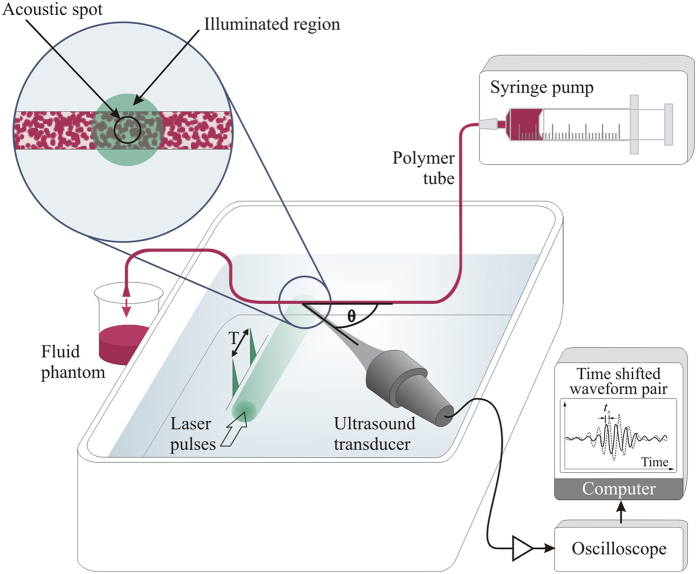
Experimental setup for pulsed photoacoustic Doppler flow measurement where the motion of micron-scale absorbing polystyrene microspheres suspended in water is used to represent blood flow. A pair of laser pulses separated by a time *T* are used to generate a corresponding pair of acoustic waveforms which are detected by an ultrasound receiver. The inset shows a typical distribution of the absorbers in the fluid phantoms. A large area (at least 5 mm diameter) of the absorbers is illuminated, but photoacoustic signals are only collected from the smaller region defined by the transducer focal spot in order to be representative of acoustic resolution PAF. The signals were amplified and then captured by an oscilloscope and downloaded to a PC, where they were processed to calculate the time shift *t*_s_ between the two waveforms that arises due to motion of the absorbers between the two laser pulses.

The results in this paper were acquired using the setup shown in Fig. 1. Pairs of photoacoustic signals were generated by exciting flowing absorbers with pairs of laser light pulses (Nano L 200-15, Litron, wavelength 532 nm) separated by a time *T* = 0.5 ms. The photoacoustic signals were detected using one or more of the three focussed ultrasound detectors listed in Table 1. The diameter of the illuminated region (at least 5 mm) was in all cases significantly larger than the diameter of the detector focal beam (less than 400 μm) in order to be representative of the acoustic resolution mode of photoacoustic detection. The angle *θ* between the transducer axis and the flow direction was measured to the nearest degree using a turntable with angular markings at 1° intervals. This estimated angle was verified by moving the tube horizontally using a translation stage, and comparing the measured distances with those calculated from the cross-correlation of photoacoustic signals acquired before and after translation.

Dark red polystyrene spheres (42922-5ML-F, Sigma-Aldrich) were used to represent red blood cells. The spheres had a diameter of 3.0 μm and were supplied in a suspension of de-ionized water with a concentration of 2.34 × 10^9 ^particles/ml. The suspension was diluted further with de-ionized water to produce a range of lower concentrations, which are described in the text as percentages of the original (100% = 2.34 × 10^9 ^particles/ml). The size of the polystyrene spheres is comparable to that of red blood cells which are typically biconcave discs about 7.5 μm in diameter and with an average thickness of 2.0 μm. The optical absorption coefficient of the spheres and the acoustic frequency spectrum they generate also match well with those of red blood cells at equivalent concentrations (see Supplementary Note 1).

Fluid flow was generated using a syringe pump (B. Braun Space^®^) with a 60 ml syringe (BD Plastipak™) and a polymer tube (Paradigm Optics). The different tubes used variously in the experiments are listed in Table 2. Measurements *V*′ ± Δ*V*′/2 were made for speeds in the range 0 to 88 mm/s. The syringe pump could be programmed to deliver flow rates in steps of 0.01 ml/hr, and the pre-selected rate and the inner diameter of the tube were used to calculate the corresponding average flow velocity *V* in mm/s. Uncertainties Δ*V* were based on the 5% tolerance in the diameter of the tubing; uncertainty due to the precision of the syringe pump was neglected since this was approximately two orders of magnitude smaller. To minimise uncertainties due to flow irregularity, the illumination region was positioned several centimetres downstream from the curved section of tube, allowing ample distance for the development of steady laminar flow^19^,^20^ at the point of photoacoustic measurement. The “known” velocity values and uncertainties *V *± Δ*V*/2 were compared with the measured *V*′ ± Δ*V*′/2 acquired via cross-correlation of the photoacoustic waveform pairs, as described in the following section.

## Signal acquisition and processing

The purpose of the signal acquisition and processing is to extract the time shift *t*_*s*_ between the two constituent signals of the photoacoustic waveform pair produced by the two excitation laser pulses. *t*_*s*_ is then used to calculate the measured velocity *V*′:


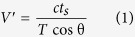


where *c* is the sound speed, *T* is the time separation between the laser pulses, and θ is the angle between the transducer axis and the flow direction. *V*′ is compared with the known velocity *V*, both of which represent the average flow velocity. Two signal processing methods based on the use of time correlation to estimate *t*_*s*_ were used as described below.

### Method 1: Cross-correlation of entire photoacoustic waveforms to give average velocity measurements

This method involves cross-correlation of the entire record length of the acquired photocoustic signals within the waveform pair in order to estimate *t*_*s*_and thus calculate the average velocity across the vessel. It is in essence the same method as that described in reference 17, but with some minor modifications. In the current studies, up to 25 waveform pairs were captured in real time and downloaded to a PC. Although in principle a velocity measurement is possible using a single pair of photoacoustic waveforms, acquisition of a train of waveform pairs reduces the effect of anomalous readings; such anomalies may arise from the random nature of the absorber distribution leading to instances of poor correlation.

**Figure 2:**
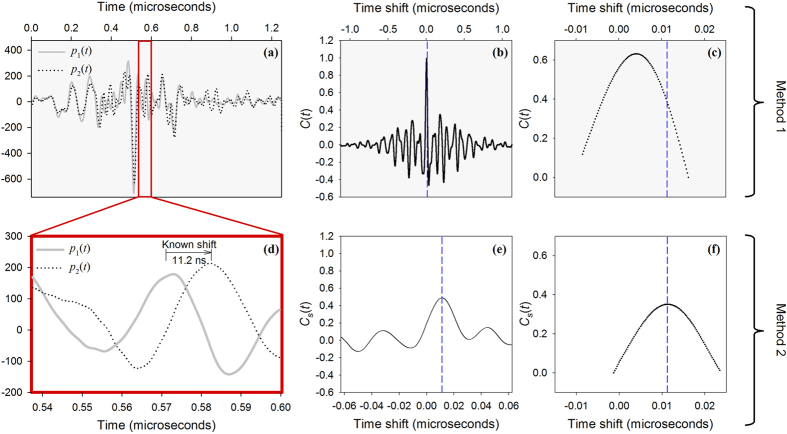
Cross-correlation of entire waveforms (top row) and waveform segments (bottom row) according to Methods 1 and 2 respectively as described in the text. (**a**) One of 25 PA waveform pairs (*p*_1_(*t*), *p*_2_(*t*), each 5000 points, 1.25 μs long), acquired using the 30 MHz transducer (θ = 45°) following double pulse illumination (*T* = 0.5 ms) of a 9% suspension of red spheres flowing at an average velocity of *V* ± Δ*V*/2 = 58 ± 6 mm/s in a 2*R* = 390 μm tube. The mean *C*(*t*) of all 25 cross-correlation functions is shown in (**b**) and an enlargement of 100 points (sampling interval 0.25 ns) centred on the peak in (**c**). 62.5 ns (250-point) segments of *p*_1_(*t*) and *p*_2_(*t*) are enlarged in (**d**), and the corresponding mean cross-correlation function *C*_s_(*t*), and 100 points around the peak are shown in (**e**) and (**f**) respectively. The dashed vertical lines in (**b,c,e,f**) mark the known time shift (11 ns). Compared to Method 1 (**c**), Method 2 (**f**) gives closer agreement between the known time shift and the measured value: the reason for this is discussed in the section on light penetration.

Figure 2(a) illustrates two photoacoustic signals (*p*_*1*_(*t*) and *p*_*2*_(*t*)) that form an example of a single waveform pair extracted from a train of 25 pairs. The discrete unbiased cross-correlation function was evaluated for the entire record length (1.25 μs) of this waveform pair, as well as for each of the other 24 pairs in the waveform train. The mean cross-correlation amplitudes at each time point were used to compute a mean cross-correlation function *C*(*t*), which is shown in Fig. 2(b); the peak of *C* was isolated and an expanded view is shown in Fig. 2(c). The maximum amplitude of a ten-point interpolant fit to this peak was used to determine the measured time shift *t*_*s*_ from which the velocity was estimated.

The final measurement *V*′ is the mean of up to twenty velocity values each calculated using 25 waveform pairs in the above cross-correlation protocol. The resolution ±Δ*V*′/2 is calculated from the standard deviation of these values: note that this error analysis is different from the method presented previously^18^ in which the resolution was obtained for a single time shift measurement (25 waveform pairs) and was equal to the uncertainty in the time shift location of the cross-correlation peak.

### Method 2: Cross-correlation of photoacoustic waveform segments to calculate velocity profiles

This method entails cross-correlation not of the entire photoacoustic waveforms as in Method 1, but rather of small segments of the photoacoustic waveforms corresponding to specific depths within the tube, in a manner analogous to “range-gating” in Doppler ultrasound. In the case where the flow velocity is not uniform across the tube diameter, it is likely that different time segments of the photoacoustic signals undergo different time shifts according to the range of flow velocities in the corresponding segments of the tube. Calculation of the average flow velocity within these smaller segments, rather than cross-correlation of the entire photoacoustic waveforms, can therefore reveal the flow velocity profile across the tube.

Figure 2 illustrates the process by which a time shift between small time segments of the photoacoustic signals can be estimated. The lower three plots, (d,e) and (f), are similar to those in (a–c), but for a small time segment (62.5 ns) of the waveform pairs. Note however that the time shift calculated using this small time segment agrees closely with the known value as shown in Fig. 2(f), whereas using the entire record length as in Method 1 produces under-reading of the known average flow velocity (Fig. 2(c)). [Fig f3]This discrepancy is discussed in further detail in the section on light penetration and laminar flow profiles.

## Results: photoacoustic velocity measurements

In order to investigate the effect of absorber heterogeneity on measurement accuracy, velocity measurements were made using different concentrations of blood-mimicking fluids comprising flowing suspensions of the 3 μm diameter red polystyrene spheres. As shown in the following section, measurement under-reading was observed and this became worse with increasing concentration. This led to further investigations of a single concentration of spheres flowing in tubes of different diameters, as presented in Figure 4.

## Absorber concentration

**Figure 3 f3:**
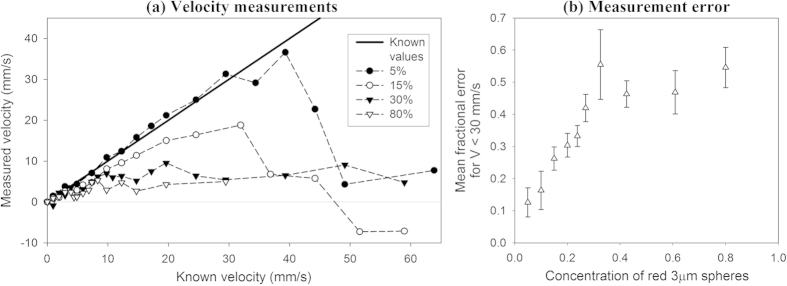
Comparison of the accuracy of velocity measurements made for suspensions of 3 μm red spheres in distilled water in concentrations ranging from 5% to 80% and flowing in a 600 μm tube. The measurements were acquired with *T* = 0.5 ms and the 80 MHz focussed transducer. (**a**) shows the measurements made for concentrations of 5%, 15%, 30% and 80% with a zero offset correction applied, and with the resolution (error bars) omitted for clarity. The mean fractional error for these and intermediate concentrations are shown in (**b**). The mean fractional error values were calculated for velocities |*V*| < 30 mm/s and the error bars represent the standard errors.

Velocity measurements were made for ten different concentrations of the polystyrene spheres ranging from 5% to 80%, and four of these data sets are shown in Fig. 3(a). For the 5% concentration of spheres, there is good measurement accuracy for known velocities up to about 40 mm/s. The measurement accuracy across the velocity range deteriorates with increasing concentration, until for the 80% concentration the velocity measurements appear to be consistently close to zero. The accuracy of each velocity measurement was calculated using the fractional difference between the known velocity (*V*) and measured value (*V*′):


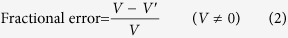


Figure 3(b) compares the mean fractional error values calculated over the velocity range |*V*| < 30 mm/s for each of the ten sets of measurements for concentrations ranging from 5% to 80%. Again, it is clear that the velocity under-reading becomes progressively worse as the concentration is increased. For the 5% concentration, the most accurate measurement in the velocity range differed from the known average flow velocity by less than 1% and the maximum resolution was 1 mm/s; for the 30% concentration a similar resolution was obtained but the majority of the measurements had a fractional error greater than 20%.

The results in Fig. 3 clearly show that the under-reading becomes worse with increasing particle concentration. This would appear to be consistent with the previously mentioned notion of heterogeneity: namely, that at high concentrations the detector bandwidth is insufficient to resolve the spatial separation of the absorbers and therefore the distribution is effectively “seen” by the detector as a static homogeneous continuum. At first sight, it might seem that the 80% suspension falls into this category given the very poor accuracy obtained at all velocities as illustrated in Fig. 3(a). However, there may be an additional contributing factor. Although increasing the particle concentration reduces heterogeneity, it also increases the light absorption. The photoacoustic signal is therefore confined increasingly to the absorbers near the wall of the tube which are slower-moving if we assume laminar flow conditions apply. If this were a significant effect, we would also expect under-reading to be exacerbated by increasing vessel diameter. This is investigated in the following section.

## Vessel diameter

**Figure 4:**
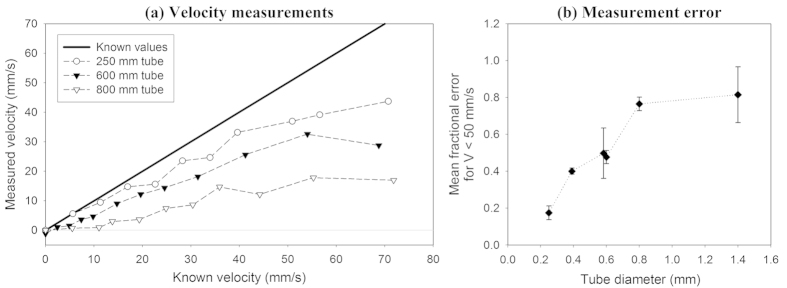
Comparison of the accuracy of velocity measurements made for a suspension of 3 μm red spheres (concentration in distilled water approximately 14%) flowing in tubes with diameters of 250 μm, 600 μm and 800 μm. The measurements were acquired with *T* = 0.5 ms and using a focussed PVDF transducer with a centre frequency of approximately 25 MHz. The velocity measurements are shown in (**a**) with the resolution (error bars) omitted for clarity. The accuracy measurements in (**b**) are the mean fractional error values calculated for all velocity measurements |*V*| < 50 mm/s and the error bars represent the standard errors.

The accuracies of the velocity measurements were compared for tubes of different diameters ranging from 167 μm to 1400 μm, each containing a flowing suspension of the 3 μm red polystyrene spheres at approximately 14%. Figure 4(a) shows velocity measurements acquired using the 25 MHz PVDF transducer (see Table 1) for three different tubes with diameters of 250 μm, 600 μm and 800 μm. It is evident that all the measurements underestimate the known velocities, and the measurement accuracy becomes worse as the tube diameter increases. Figure 4(b) shows the mean fractional error values calculated over the velocity range |*V*| < 50 mm/s for each set of measurements acquired for the different tube diameters. Again, it is clear that the measurement accuracy deteriorates (the fractional error increases) as the tube diameter is increased.

As suggested in the previous section, these results imply that, as well as insufficient absorber heterogeneity, limited light penetration which biases the measurement towards slow-moving absorbers close to the tube wall may also be a contributing factor to the under-reading observed in Fig. 3. In order to explore the relative contributions of both influences in more depth, the following two sections consider each individually.

## Absorber heterogeneity and photoacoustic signal bandwidth

As discussed previously, accurate measurement of time shifts by tracking a unique photoacoustic signature requires the absorbers to be distributed with sufficient spatial heterogeneity; otherwise, in a homogeneous medium, the motion is indistinguishable from the static case. “Heterogeneity” in this context is a loose term referring to the degree of non-uniformity in the absorbing medium as perceived by the detector: for example, a suspension of absorbing microspheres that appears “homogeneous” using a narrowband 5 MHz detector may appear “heterogeneous” using a broadband 30 MHz detector. In order to acquire a deeper understanding of heterogeneity and its influence on velocity measurement accuracy, we consider the following: first, relationship between the absorber distribution and the acoustic frequency content of the generated photoacoustic wave; second, the way in which the detector modifies this frequency content and finally its impact on velocity measurement accuracy.

**Figure 5:**
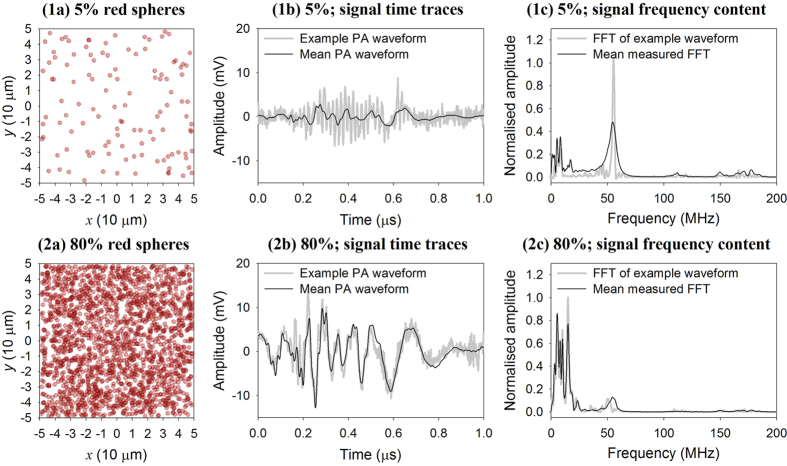
Comparison of photoacoustic (PA) signal time series (b) and normalised signal frequency content (c) for a low concentration (5%, first row) and a high concentration (80%, second row) of the red spheres. The plots in (**1**a) and (**2**a) show two-dimensional views of simulated 3 μm diameter spheres distributed at 5% and 80% concentrations respectively (average particle separations 26.1 ± 0.2 μm in and 8.42 ± 0.01 μm: see equation for Wigner-Seitz radius in Supplementary Information online) in a cube with a side length of 100 μm. The photoacoustic data in (b) were acquired using the 80 MHz transducer and for suspensions flowing in a 600 μm tube. The waveforms shown in black are the mean of over 2000 PA signals acquired for velocities ranging from 0 mm/s to 80 mm/s, and the example waveforms shown in grey are those with the median amplitude. The normalised frequency spectra in (c) are fast Fourier transforms (FFTs). The black line shows the mean of over 2000 FFTs calculated for the same set of signals that are evaluated in (b); the grey line is the FFT of the example signal shown in (b).

**Figure 6:**
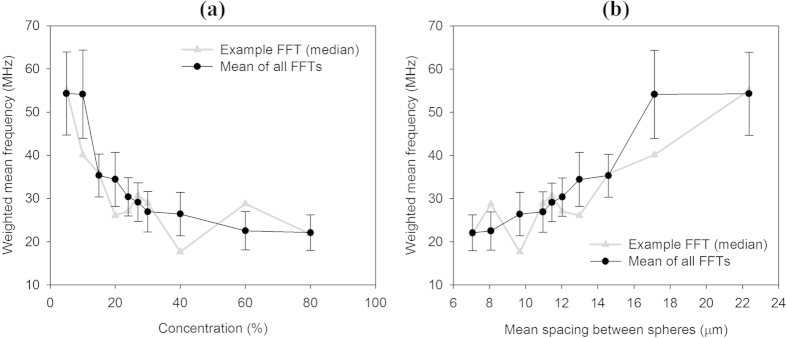
(**a**) Weighted mean frequencies (WMFs) calculated from the fast Fourier transform (FFT) of signals acquired for different concentrations of 3 μm spheres. The WMF was calculated by summing the product of the amplitudes and the frequencies and normalising by the sum of the amplitudes. The black data points show the mean WMF of over 2000 FFTs calculated for a set of the same number of PA signals acquired for the relevant concentrations, and the error bars represent the standard deviation. The grey data points show examples of WMFs calculated from a single PA signal of median amplitude. Examples of the mean and median FFTs for the 5% and 80% concentrations are shown in Fig. 5. (**b**) Weighted mean frequencies as in (**a**) but plotted vs. the mean spacing between the spheres, calculated from the Wigner-Seitz radius which assumes a random distribution (see Supplementary Information online).

Figure 5 shows the time series and corresponding frequency spectra of measured photoacoustic signals acquired using the 5% and 80% suspensions. In plots (1b) and (2b), an example of an individual photoacoustic (PA) waveform is shown with the mean of all the PA signals overlaid. In plots (1c) and (2c) the frequency spectrum corresponding to the individual PA signal is overlaid by the mean of all the frequency spectra. Both plots show a peak in the spectra at about 60 MHz, which is a feature of the detector frequency response. However, it is evident that for the 80% concentration this peak is significantly lower in amplitude and there is a larger proportion of lower frequency components present. This trend of downshifting in the acoustic frequency content with increasing concentration is further illustrated in Fig. 6 which shows the weighted mean frequency of the acoustic spectrum for different concentrations ranging from 5% to 80% in (a) and the same values are plotted in (b) for the corresponding mean particle separations calculated using the Wigner-Seitz radius (see Supplementary Information, Equation 1). This behaviour is consistent with numerical simulations carried out using the k-Wave MATLAB toolbox^21^ (see Supp. Figs. S3 and S4) which also show the signal frequency content being progressively downshifted to lower frequencies with increasing concentration.

Certainly, a reduction in high frequency content seems plausible at the maximum possible packing density when the absorbing particles have effectively coalesced into a continuum (bottom row in Supp. Fig. S3). Under these conditions the suspension represents a spatially homogeneous absorber. The acoustic frequency content is now no longer defined by the micron scale absorbing particles but by the bulk absorption of the relatively large 100 μm sided cuboidal domain and thus downshifted relative to that at low concentrations. However, the simulation results in Supp. Figs. S3 and S4 and the experimental data in Fig. 6 show this frequency downshifting also occurring for suspensions with concentrations well below the maximum packing density even though these suspensions are clearly still spatially heterogeneous. This is perhaps surprising since the simulated signals in Supp. Fig. S3 are not significantly bandlimited by the computational grid; it might therefore be expected that arbitrarily high concentrations up to (but not including) the maximum packing density would retain their high frequency content and thus be perceived by the detector as heterogeneous.

Bandlimiting due to intrinsic detector frequency response is insufficient to explain the above-mentioned frequency downshifting. Instead, the explanation for this unexpected behaviour is related to the field-of-view of the detector, which results in spatial averaging of the absorber distribution. The simulated signals in Supp. Fig. S3 are recorded by point-like omnidirectional detectors. The detected photoacoustic waveform therefore represents a set of time-retarded projections of the mean value of the initial pressure distribution p_0_ integrated over a series of spherical surfaces of increasing radius centred on the detector. As the concentration increases, fluctuations in the mean value of p_0_ for successive spherical surfaces reduce and eventually assume a near constant value. The detected photoacoustic waveform thus begins to resemble that produced by a large homogenous absorber rather than an ensemble of microscopic spheres. The signal frequency content is therefore downshifted and the absorber is perceived as “homogenous” by the detector even though its bandwidth is infinite. Contrast this with a hypothetical perfectly directional detector – again, with infinite bandwidth – that receives signals only along an infinitesimally narrow column along its line of sight. Under these conditions, the above-mentioned spatial averaging effect does not occur. There is therefore no frequency downshifting and arbitrarily high concentrations up to the maximum packing density will be perceived as heterogeneous by the detector. Of course, once the maximum packing density is reached, the absorber is truly homogeneous and downshifting does then abruptly occur. This behaviour is illustrated in Supp. Fig. S5 which shows the simulated time resolved signals and corresponding frequency spectra for different particle distributions for a directional detector. Supp. Fig. S6 provides a compelling illustration of the abrupt transition to the homogenous case as evidenced by the rapid fall in the weighted mean frequency at the point (*N* = 23 spheres per 100 μm) at which the particles begin to coalesce. By contrast the spatial averaging effect of the omnidirectional detector produces a gradual reduction in weighted mean frequency with increasing concentration as illustrated in Supp. Fig. S4 signifying a progressive transition to perceived homogeneity. The significance of these observations is that they suggest that the concept of heterogeneity in the context of AR-PAF is rather more subtle than expected in that a suspension can be rendered effectively homogeneous not only by the limited bandwidth of the detector but also by its finite field-of-view. Both effects will compromise the ability to make accurate flow measurements.

In the case of the focused detectors used in this study, the situation lies somewhere between the above two extremes. A focused detector is neither perfectly omnidirectional nor directional but spatially averages over cross sectional surfaces at different depths within its focal region. Downshifting of the signal due to spatial averaging will, to some extent, therefore still occur. Indeed this appears to be responsible for the dominance of the low frequency content for the 80% suspension, compared to the 5% suspension shown in Fig. 5. Further evidence for downshifting due to spatial averaging over the detector field of view is provided in Fig. 6 which shows the progressive reduction in the weighted mean frequency with increasing concentration in qualitative agreement with the simulations in Supp. Fig. S4. However, although spatial averaging may be one reason for the inability to measure flow in the 80% suspension as illustrated in Fig. 3, it should also be noted that the mean separation of the individual microspheres at 80% concentration corresponds to frequencies of the order of 200 MHz. This is well beyond the 80 MHz detector bandwidth so in this case, even in the absence of spatial averaging, the high concentration of microspheres would likely be perceived by the detector as homogeneous thereby precluding velocity measurement.

**Figure 7:**
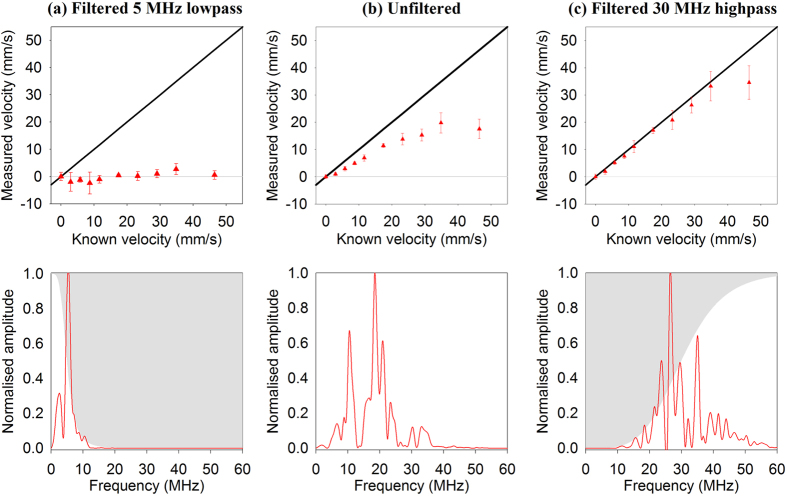
The effect of low-pass and high-pass filtering the photoacoustic data on velocity measurements made for a 9% concentration of red spheres flowing in a tube of diameter 390 μm. The PA signals were acquired using a PZT transducer with a centre frequency of 30 MHz. The velocity measurements for the unfiltered data are shown in (**b**) and the normalised mean FFT of all the signals is shown below. The measurements and corresponding FFT spectra in (**a**) and (**c**) are for PA signals after they were passed through a low-pass 5 MHz filter (**a**) or a high-pass 30 MHz filter (zero-phase Butterworth filter, order 2).

**Figure 8:**
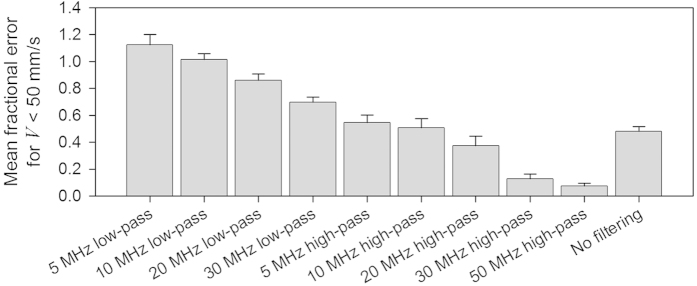
Comparison of the accuracy of velocity measurements after passing the PA signals through one of various low-pass and high-pass filters. The velocity measurements were acquired using the 30 MHz transducer (θ = 45°) following double pulse illumination (*T* = 0.5 ms) of a 9% suspension of red spheres flowing in a 2*R* = 390 μm tube. The mean fractional error values were calculated for velocities |*V*| < 50 mm/s and the error bars represent the standard errors.

Implicit in the above description is the notion that heterogeneity is signified by the presence of high frequencies in the detected photoacoustic signal; this facilitates the measurement of smaller time shifts and is thus conducive to making accurate velocity measurements. By contrast, the dominance of low frequencies due to detector bandlimiting or spatial averaging or a combination of both implies reduced perceived heterogeneity and thus limited measurement accuracy. In order to illustrate the importance of high-frequency signal content to the accuracy of the velocity measurements, photoacoustic signals obtained in a 9% suspension were passed through different frequency filters. Figure 7(a) shows that passing the photoacoustic signals through a low-pass filter exacerbates the measurement under-reading; on the other hand, the results shown in Fig. 7(c), where the signals were passed through a high-pass filter, show improved measurement accuracy compared to the unfiltered case (b). Thus, at low concentrations as in this case where spatial averaging due to the finite detector field-of-view does not cause significant downshifting, the perceived absorber heterogeneity is strongly related to the detector bandwidth alone, with detection of high frequencies clearly necessary for accurate velocity measurements. In order to further illustrate this point, Fig. 8 compares the accuracy of velocity measurements calculated from photoacoustic signals with different degrees of high and low pass filtering applied. Whilst low-pass filtering is clearly detrimental to velocity measurement accuracy, high-pass filtering enables recovery of the average flow velocity (as also shown in Fig. 7). Note also that it is not solely the presence of high frequencies that is required for accurate measurements. The absence of the low frequencies also appears to be necessary; for example, the 10MHz and 20MHz high pass filtered signals yield higher accuracy than that obtained using the 30MHz low pass filter. This is most likely because the low frequency components of the spectrum are associated with the high amplitude part of the time-domain signal. The latter dominates the cross correlation reducing the influence of the high frequency components on to which the time shift information is predominantly encoded.

## Light penetration and laminar flow profiles

Adequate absorber spatial heterogeneity is certainly a requirement for accurate velocity measurements as discussed in the previous section. However, the effect of increasing tube diameter on measurement under-reading, as shown in Fig. 4, highlights another consideration involving the light penetration into the tube relative to the flow profile.

Within the tube the microspheres are flowing in a laminar regime. This is evident from the Reynolds number Re, which is given by


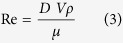


where *D* is the tube diameter, *V* is the average flow velocity in the tube, ρ is the density of the flowing fluid and μ is the dynamic viscosity of the flowing fluid. Even in the “worst” experimental case, the value calculated for Re is an order of magnitude smaller than the critical Reynolds number of 2000 beyond which there is a transition to turbulent flow.

The laminar velocity profile 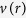 in a tube of radius *R* can be derived^22^,^23^ from the Navier-Stokes equations to give:


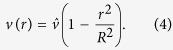


At a distance *r* = 0 from the central axis the velocity is maximum, and denoted 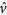. By computing the integral of 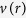, giving the Hagen-Poiseuille’s law for flow rate, it can be shown that the average flow velocity 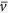 across the tube cross-sectional area is half the maximum velocity 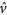 at the centre. This average velocity 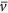 is the value denoted by the “known” velocity *V* with which the photoacoustically measured velocity *V*′ is compared in the flowmetry experiments.

**Figure 9:**
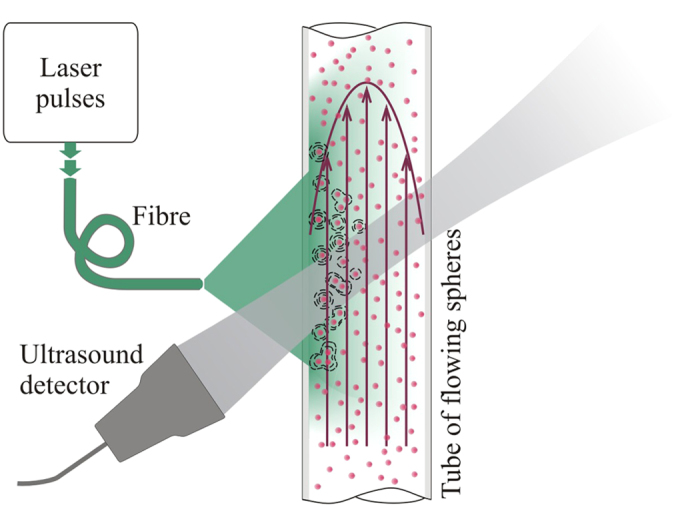
Illustration of the effect of laminar flow and light attenuation, resulting in generation of the largest amplitude photoacoustic signals in slow-moving absorbers at the near edge of the tube.

Given that the flow in the tube is laminar with a parabolic flow profile, in the presence of strong light attenuation the photoacoustic signal is generated predominantly from the slower-moving absorbers close to the tube wall. This effect is illustrated in Fig. 9. As the absorption increases, the photoacoustic signal generation becomes increasingly confined to the absorbers close to the edge of the tube. This explains why the measurement under-reading in Fig. 3 becomes worse with increasing concentration. In addition, as the tube diameter is increased, the light penetration is smaller relative to the tube radius, and thus there is greater measurement under-reading even if the absorption remains constant. This explains the results in Fig. 4 where the accuracy becomes worse with increasing tube diameter.

The under-reading caused by the light attenuation can be addressed by analysing small segments of the photoacoustic waveforms corresponding to specific sections across the tube diameter, using the range-gating method described previously in this paper (Method 2). Figure 2 illustrates the extraction of the average flow velocity using segments of a single waveform pair. In Fig. 2(f), the peak of the mean cross-correlation function *C*_*S*_(*t*) occurs at a horizontal position that coincides almost exactly with the “known” (average) time shift (*t*_s_ = 11 ns), whereas the peak value of *C*(*t*), shown in Fig. 2(c) for the entire waveforms, occurs at a time shift of 4 ns and thus significantly under-reads the known value. This suggests that the time shift calculated from the entire waveforms is indeed biased towards the slower velocities in the flow profile, whereas by analysing only a small waveform segment, the average velocity can be more accurately quantified. This is because the waveform segment is chosen such that it reflects velocities deeper in the tube rather than at the edge where the velocity is low.

**Figure 10:**
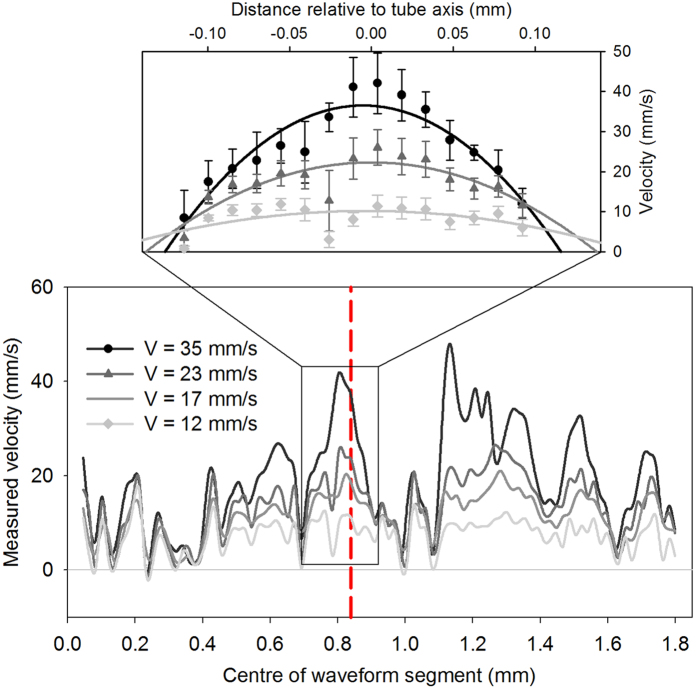
Profiles of velocity measurements corresponding to signal segments shifted progressively along the photoacoustic signal time axis by 50 points (12.5 ns, or 18.45 μm). The measured velocity (*y* axis) is the mean value calculated for six individual velocity measurements acquired using the 30 MHz transducer (θ = 45°) following double pulse illumination (*T* = 0.5 ms) of a 9% suspension of red spheres flowing in a 2*R* = 390 μm tube. The dashed line shows the segment for which the velocity measurements represent the average flow velocity, as illustrated in Fig. 11. This part of the profile corresponds to the flow within the tube and is enlarged to illustrate laminar (parabolic) fits to the data points.

**Figure 11:**
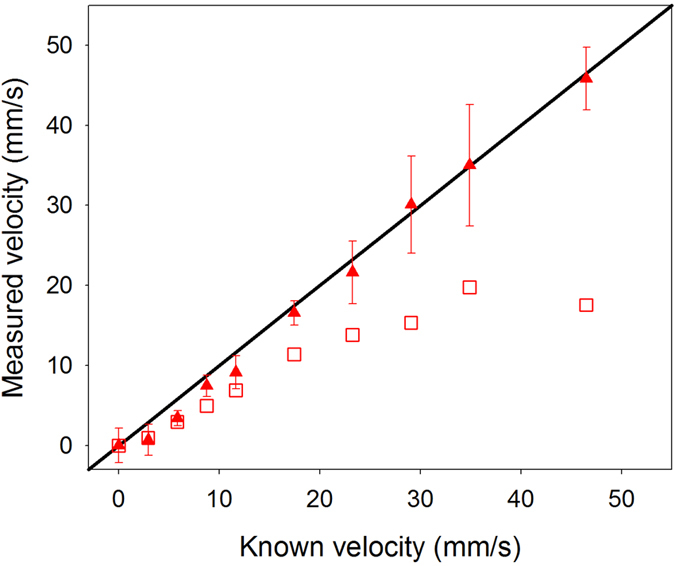
Mean velocity measurements made for the segment marked by the red dashed line in Fig. 10, acquired using Method 2 described in connection with Fig. 2. For comparison, the unfilled squares show the original data that was acquired using Method 1 and shown previously in Fig. 7(b).

In fact, the range-gating method can be generalised beyond the specific example illustrated in Fig. 2. Figure 10 illustrates the most general approach in which estimation of the time shifts for multiple successive waveform segments in the photoacoustic signal, corresponding to different distances across the tube, allows the spatial profile of velocity to be mapped. If required, this velocity profile can also be used to extract a single representative measure, for example of the average flow velocity. Figure 11 shows that, using this approach, it is possible to accurately recover the known average flow velocity for measurements in the velocity range *V* = 0 to 50 mm/s from the velocity profile. The mean fractional error of the measurements in this range is less than 19%, which is a considerable improvement compared to the original data shown in Fig. 7(b); the latter demonstrates significant under-reading of the known average flow velocity with a mean fractional error of approximately 48%. This accuracy is comparable to that obtained by high pass filtering of the original data, as shown in Fig. 7(c). However, in general range-gating is a more valuable approach compared to filtering since the former also allows mapping of velocity profiles across the tube.

## Conclusions

In this study, acoustic resolution photoacoustic Doppler velocimetry (AR-PAF) was demonstrated using carefully controlled blood-mimicking fluid phantoms comprising suspensions of 3 μm diameter red polystyrene spheres, which are similar in size and optical properties to red blood cells. Velocity measurements were made for different concentrations of the spheres, and for different tube diameters. The results have shown that under some circumstances the technique can accurately measure flow with a resolution of approximately 1 mm/s, which would be sufficient for blood flow measurement in microvasculature (typically less than 50 mm/s). However, adequate velocity measurement accuracy (<10% fractional error) was only obtained for concentration less than about 2.3 × 10^8 ^spheres/ml, which is well below the physiologically realistic concentrations of red blood cells (approximately 4 × 10^9 ^cells/ml) in whole blood. At higher concentrations, consistent measurement under-reading was observed which was exacerbated by increasing concentration (Fig. 3) and by larger tube diameters (Fig. 4). The source of these errors was found to be related to the absorber heterogeneity and the light penetration within the tube. Both influences were investigated individually.

Figure 9 illustrates how exponential decay of light intensity across the tube can lead to a measurement bias towards the slower-moving absorbers at the edge of the tube. This bias is intensified as the light penetration reduces relative to the tube diameter, which is consistent with the observation that the under-reading of the average flow velocity becomes worse with increasing absorber concentration (Fig. 3) and increasing tube diameter (Fig. 4). In these experiments, the problem of limited light penetration was aggravated by the illumination wavelength of 532 nm, which is strongly absorbed by the microspheres. In practice, blood velocity measurements would be carried out using more deeply penetrating near-infrared light (typically in the range 670 nm–900 nm) at a wavelength which provides sufficient penetration whilst retaining adequate SNR. In order to reduce the measurement bias towards the absorbers close to the edge of the tube, a “range-gating” method was used. Figure 11 shows that this can significantly improve the velocity measurement accuracy relative to the average flow velocity. More importantly, range-gating enables mapping of the velocity profile across the vessel as shown in Fig. 10, which is advantageous for studying the dynamics of blood flow. Indeed this is the most general approach to PAF, although the average flow velocity or some other representative metric can still be extracted from the profile if required.

Aside from the need for sufficient light penetration, a more fundamental consideration is the requirement for adequate absorber heterogeneity. A suspension of absorbers is deemed heterogeneous if they can be resolved using the detector. Conventionally, it is thought that this resolving ability is solely a function of the intrinsic bandwidth of the detector: bandlimiting by the detector will result in detection of only the low frequency content of the generated photoacoustic signal thus causing the suspension to appear less heterogeneous and compromising the velocity measurement accuracy. This notion is supported by Figs 7 and 8 where low pass filtering results in poor measurement accuracy, but high pass filtering provides high accuracy, demonstrating that, as expected, it is the high frequency content and not the low frequencies that are conducive to accurate measurements. However, this study has also shown that this conventional understanding of heterogeneity is rather more nuanced than previously articulated. As well as the above intrinsic detector bandlimiting, there is a second influence due to the detector that effectively causes bandlimiting and reduces heterogeneity in the same way. This effect is the spatial averaging that occurs over the detector field-of-view as described in the section on absorber heterogeneity and can result in a suspension being perceived as homogeneous even when the detector bandwidth is infinite. Evidence for this is seen in the experimental data in Figs 5 and 6, and also in the numerical simulations in the Supplementary Information (Figs. S3 and S4) which show the frequency content of the photoacoustic signals being progressively downshifted with increasing absorber concentration. Thus, in order to make accurate velocity measurements, it is necessary to avoid both effects: first, the intrinsic bandwidth of the detector must be sufficiently wide that it can detect wavelengths comparable to the spatial separation of the absorbers, and second the detector field-of-view should be sufficiently narrow such that excessive spatial averaging over the absorber distribution does not occur. Both requirements become increasingly challenging with increasing absorber concentration.

The two-fold bandlimiting effect described above has profound implications for AR-PAF in whole blood. Using the 3 μm microspheres and focussed transducers with bandwidths of several tens of MHz, it was found that accurate velocity measurements were possible using suspensions of concentrations up to 10% (2.3 × 10^8 ^spheres/ml) where the estimated particle spacing corresponds to frequencies of up to approximately 90 MHz. By contrast, the 80% suspension (1.9 × 10^9 ^spheres/ml) corresponds to over 200 MHz and provided poor measurement accuracy. Since a physiologically realistic concentration of red blood cells is 4 × 10^9 ^cells/ml, we would therefore expect accurate flow measurements in whole blood to demand detection of frequencies in the hundreds of MHz range. However, tissue penetration depth at these very high frequencies would be very low (<0.5 mm) due to frequency dependent acoustic attenuation thereby removing any advantage over OR-PAF. As well as an impractically large detector bandwidth, a very narrow detector field-of-view would also be required in order to minimise bandlimiting due to spatial averaging. However, this in turn risks compromising velocity range, resolution and accuracy since the time separation *T* between the excitation laser pulses would need to be reduced in order to track the red blood cells before they move outside the small field of view.

Although these factors suggest that making accurate AR-PAF measurements in blood is likely to be non trivial, it may well prove to be less difficult than expected. Based on the above calculations for a non-aggregating randomised distribution of red blood cells it would seem that AR-PAF measurements in blood would require an impractically large detector bandwidth and narrow field of view. However, it is well known that red blood cells can form clusters, aggregates or rouleaux thereby increasing heterogeneity offering the prospect of making flow measurements using lower frequency detectors. Indeed, it has been shown that, whilst photoacoustic signals from non-aggregated red blood cells are dominated by frequencies greater 200 MHz, under physiologically realistic conditions of red blood cell aggregation the frequency content is reduced to a few tens of MHz.^24^. This suggests that the heterogeneity requirement might be fulfilled, at least partially, by detectors with bandwidths at these lower frequencies rather than in the hundreds of MHz range. Moreover encouragement may be taken from the success of pulsed wave Doppler ultrasound^6^ and cross-correlation pulse-echo ultrasound measurement of blood flow^25^, which would appear, at least in principle, to have a comparable heterogeneity requirement to that for AR-PAF but can utilise detectors of only a few MHz. One factor that may preferentially assist pulsed Doppler ultrasound however is the presence of speckle which is rarely observed using photoacoustic methods.

In summary, this work has provided new insight into the factors that affect AR-PAF, namely the light penetration and the two-fold detector bandlimiting. These findings will help direct the choice of appropriate illumination and detection parameters for measurements in blood, which so far have proved challenging in the acoustic-resolution mode. Further investigation of these challenges will entail experiments using near-infrared wavelengths of light that are less attenuated thereby alleviating the problem of average velocity underestimation due to limited light penetration. Also, the perceived heterogeneity of the absorbers will be further explored using different red blood cell concentrations, and various detector frequencies and bandwidths. These future investigations may pave the way to realising the potential of AR-PAF for making deep tissue flow measurements in the microvasculature.

## Additional Information

**How to cite this article**: Brunker, J. and Beard, P. Acoustic resolution photoacoustic Doppler velocimetry in blood-mimicking fluids. *Sci. Rep.* **6**, 20902; doi: 10.1038/srep20902 (2016).

## Supplementary Material

Supplementary Information

## Figures and Tables

**Table 1:** Transducers used variously to detect photoacoustic signals generated in the light-absorbing phantoms.

Centrefrequency (MHz)	Transducermaterial	Focallength(mm)	FWHM beamwidth (mm)
30	PZT	19	0.35
25	PVDF	24	0.24
80	PVDF	12	0.0075

The 30 MHz transducer (part number: V375) was manufactured by Panametrics Olympus NDT Inc. (Waltham, Massachusetts, USA), the 25 MHz transducer was made by Precision Acoustics Ltd. (Dorchester, UK) and the 80 MHz transducer was made by Krautkramer Foertser Co. Ltd. (Tokyo, Japan). The full-width-half-maximum (FWHM) values were calculated from the transducer characteristics.

**Table 2:** Nominal values for the inner diameters (I.D.) of various polymer tubes (Paradigm Optics) used to contain the flowing absorbers.

Material	I.D. (μm)
THV-500	167
PMMA (acrylic)	250
THV-500	390
THV-2030G	583
PMMA (acrylic)	600
THV-2030G	800
PMMA (acrylic)	1400
