# Erratum: Acoustic resolution photoacoustic Doppler velocimetry in blood-mimicking fluids

**DOI:** 10.1038/srep23881

**Published:** 2016-04-20

**Authors:** Joanna Brunker, Paul Beard

Scientific Reports 6: Article number: 2090210.1038/srep20902; published online: 02192016; updated: 04202016.

The original version of this Article contained errors.

In the Introduction section,

“This time correlation AR-PAF approach has previously been shown to provide a high level of quantitative accuracy (<1%) when using a solid moving phantom comprising a distribution of micron-scale absorbing features printed on to a polymer sheet^19^.”

now reads:

“This time correlation AR-PAF approach has previously been shown to provide a high level of quantitative accuracy (<1%) when using a solid moving phantom comprising a distribution of micron-scale absorbing features printed on to a polymer sheet^18^.”

In the Acoustic resolution photoacoustic Doppler velocimetry section,

“Velocity measurements were made in fluids using a time-correlation Doppler flowmetry approach, which is described in detail in reference 17.”

now reads:

“Velocity measurements were made in fluids using a time-correlation Doppler flowmetry approach, which is described in detail in reference^18^.”

In the Signal acquisition and processing section under subheading ‘Method 1: Cross-correlation of entire photoacoustic waveforms to give average velocity measurements’,

“It is in essence the same method as that described in reference 17, but with some minor modifications.”

now reads:

“It is in essence the same method as that described in reference^18^, but with some minor modifications.”

The Acknowledgements section in this Article was incomplete,

“This work was supported by the UK Engineering and Physical Sciences Research Council (EPSRC) and European Union project FAMOS (FP7 ICT, Contract 317744). The authors would also like to thank Litron for the use of their laser, Tom Millard for providing MATLAB code to simulate random distributions of spheres, and Ben Cox for reading the manuscript.”

now reads:

“This work was supported by the UK Engineering and Physical Sciences Research Council (EPSRC) and European Union project FAMOS (FP7 ICT, Contract 317744). The authors would also like to thank Litron for the use of their laser, Nader Saffari for the use of his ultrasound transducers, Tom Millard for providing MATLAB code to simulate random distributions of spheres, and Ben Cox for reading the manuscript.”

These errors have now been corrected in the PDF and HTML versions of the Article.

In addition, an incorrect Supplementary Information file, which omitted the Supplementary References, was inadvertently published with this Article. The correct Supplementary Information now accompanies the Article.

