# Supplementary material for: Acoustic resolution photoacoustic Doppler velocimetry in blood-mimicking fluids
Source: Sci Rep. 2016 Feb 19;6:20902. doi: 10.1038/srep20902 (PMC4759580; doi:10.1038/srep20902)
Supplement: Supplementary Information [file srep20902-s1.pdf]

Acoustic resolution photoacoustic Doppler velocimetry in blood-mimicking fluids

Joanna Brunker[1,]*, Paul Beard[1]

Supplementary Information

[1] Department of Medical Physics and Biomedical Engineering, University College London,

Gower Street, London WC1E 6BT, UK

* Corresponding author. Tel.: +44 (0)20 7679 0455; E-mail: joanna.brunker.09@ucl.ac.uk

Supplementary Note 1: Comparison between polystyrene spheres and red blood cells

The optical properties of the two materials are closely similar, as are the photoacoustic signals they emit, which suggests that the polystyrene spheres are highly representative of red blood cells.

First consider the optical absorption coefficients. These were measured for both the polystyrene spheres and red blood cells at a concentration of approximately 2×10^{8} ml^{-1}. Supplementary Figure S1 shows measurements of absorption coefficients for wavelengths between 500 nm and 550 nm. At the laser wavelength of 532 nm, which was used in the flowmetry experiments, the absorption coefficients for the two suspensions differ by less than 10%.

Subsequent experiments compared the photoacoustic signals emitted by the two suspensions when contained within 390 µm tubes and exposed to a pulsed laser beam. A 30 MHz transducer was used to detect signals first from the suspension of red blood cells and second from the suspension of red polystyrene spheres. The individual signal time traces and frequency contents for over 4,750 photoacoustic waveforms, as well as the average values, were compared between the suspensions. Supplementary Figure S2 shows that the time resolved signals and frequency content of the two suspensions are similar.

Using these microspheres rather than blood has the advantage that they are reproducible and stable over time, allowing many repeated measurements under identical conditions with precisely defined experimental parameters. This enables a systematic and rigorous investigation of all the relevant factors affecting accuracy to be undertaken in a highly controlled manner in a way that would be difficult to achieve with blood given its physical and chemical variability, limited lifetime and availability restrictions.

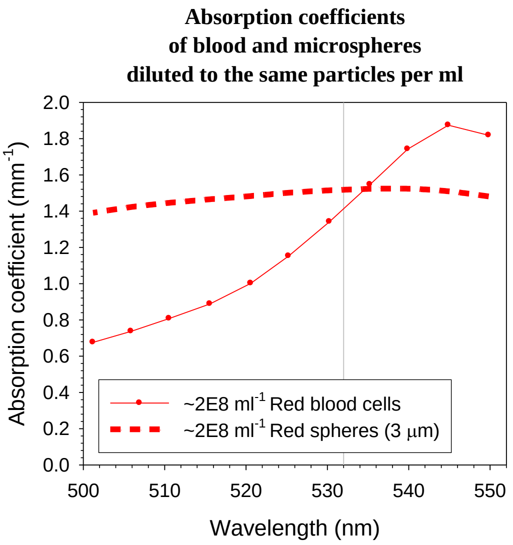

Supplementary Figure S1 Absorption coefficient spectra for red blood cells and red polystyrene microspheres (42922-5ML-F, Sigma-Aldrich). The absorption coefficient for each suspension was obtained by fitting an exponential function to the detected photoacoustic waveform[25]. The particle concentrations were approximately 2x10^{8} ml^{-1}. A vertical line marks 532 nm, which was the visible laser wavelength used in the flowmetry experiments.

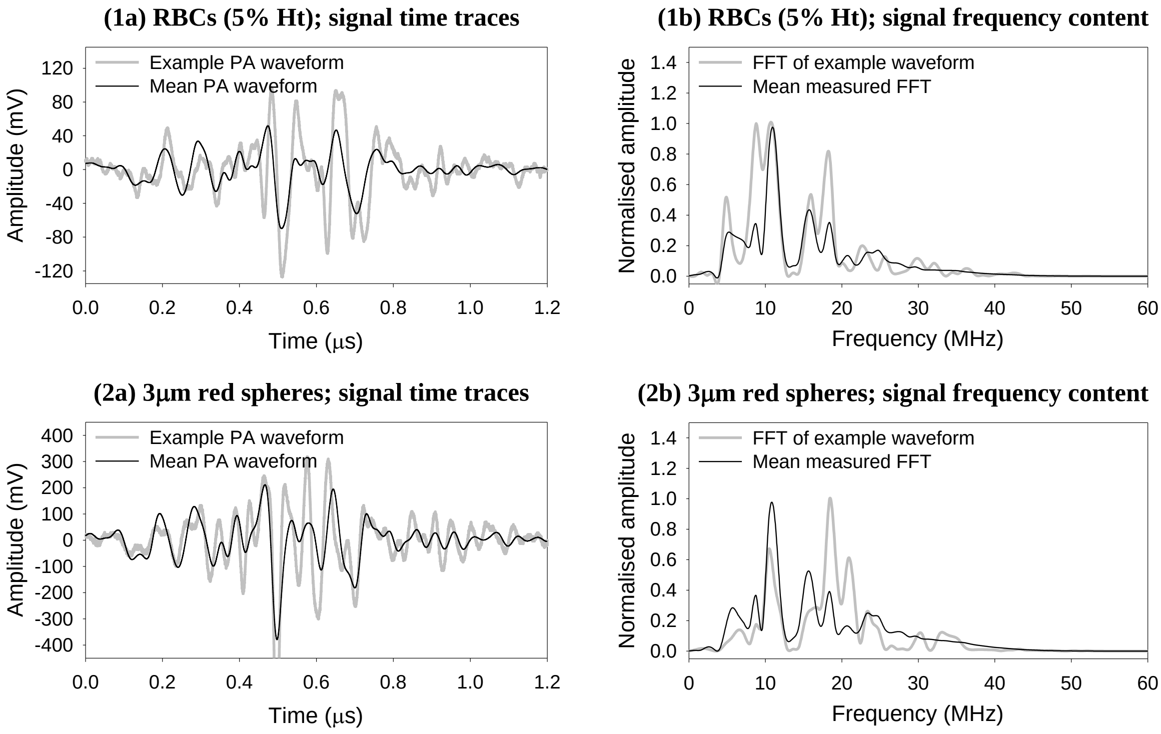

Supplementary Figure S2 Signal waveforms (a) and frequency spectra (b) corresponding to velocity measurements acquired for red blood cells (RBCs) and red polystyrene spheres (3 μm), both made to a concentration equivalent to approximately 5% Ht. The waveforms and frequency spectra are the means of over 4,750 photoacoustic waveforms acquired with the 30 MHz focussed transducer. The example waveforms shown in grey are those with the median amplitude.

Supplementary Note 2: Absorber heterogeneity

The relative heterogeneity of different suspensions can be estimated using the average particle spacing, g, which can be calculated from simulations of random distributions of spheres. Plots (1a) and (2a) in Figure 5 show two-dimensional views of simulated distributions of spheres in a cube with a side length of 100 µm: the simulation in (1a) is for a random distribution of 3 µm diameter spheres at the 5% concentration used in the experiments, and (2a) shows the 80% concentration. The mean distance calculated between adjacent spheres is g = 26.1 ± 0.2 µm in (1a) and g = 8.42 ± 0.01 µm in (2a). These values agree reasonably well with those of g' = 22.36 µm and g' = 7.06 µm calculated from the Wigner-Seitz radius r_s [26], which is defined for a random distribution of N non-aggregating particles contained within a volume V; each particle occupies a spherical region with a radius equal on average to r_s:

$$r_s = \left(\frac{3V}{4\pi N}\right)^{1/3}. \tag{1}$$

If each particle has a radius r ($2r$ = 3 µm), the mean separation g' is:

$$g' = 2(r_s - r). \tag{2}$$

Numerical simulations were carried out in order to investigate further how the photoacoustic signal frequency content is determined by the spatial characteristics of the absorber distribution.

Simulations for omnidirectional detectors

First, an initial pressure distribution was simulated using uniformly absorbing spheres with a diameter of 3 μm randomly distributed within a cube of side length 100 μm. At a distance of 10 μm from the cube, there were 21 pixels designated to represent a linear array of omnidirectional point detectors. The acoustic propagation of the pressure waves to these detectors was simulated using a pseudospectral acoustic model implemented using the k-Wave MATLAB toolbox[20]. The "smooth" function was applied to the initial pressure distribution and the pressure waves were propagated through a three-dimensional non-absorbing homogeneous acoustic medium.

The results of the simulation are shown in Supplementary Figure S3. The first row in Supplementary Figure S3 shows the results for a single sphere. The photoacoustic waveforms (column b) are each a characteristic "N" shape, as expected from the analytical solution[27], and the signal extends over a broad range of frequencies up to about 500 MHz (column c). The addition of a second sphere into the domain introduces a second N-shape pulse into the photoacoustic time trace. The FFT shows the same broad envelope as for the single sphere, still with maximum amplitude around 160 MHz, but with additional structure due to the temporal separation between the two time domain signals. The broadband envelope is still discernible for the 5% distribution of spheres (third row in Supplementary Figure S3), but there is a slightly larger component of frequencies less than 50 MHz. The latter is a consequence of spatial averaging over the absorber distribution imposed by the detector field-of-view as described in Section 4. This spatial averaging effect is even more significant for the 80% distribution of spheres (fourth row in Supplementary Figure S3) where the frequencies less than 10 MHz dominate the other spectral peaks. The extreme case of spatial averaging is represented when the spheres are so tightly packed that they effectively coalesce into a homogenous cube (fifth row in Supplementary Figure S3) which produces a dominant peak around 6 MHz, and frequency components greater than about 200 MHz are negligible.

In order to compare the frequency content of signals simulated for various different concentrations of spheres, the FFTs were characterised using a weighted mean value. This was calculated by summing the product of the amplitudes and the frequencies, and normalising by the sum of the amplitudes. This was repeated for each of the signals received at the 21 simulated sensors, and a mean and standard deviation of these 21 values were calculated. Supplementary Figure S4 (a) shows the weighted mean frequencies (WMFs) for FFTs corresponding to spheres distributed at concentrations ranging from 5% to 100% of the red sphere concentration used in the experiments (2.34 x 10^9 particles/ml). The WMF values are also shown for 240%, which corresponds to spheres spaced on average by g = 4.7 µm, and for a solid cube, which represents the limiting case where the particle spacing is effectively zero. Supplementary Figure S4 (b) also shows the WMFs for concentrations of 3% and 1%, and for the 2 spheres shown in the second row of Supplementary Figure S3, and these correspond to g = 33 µm, 43 µm, and 54 µm respectively. For mean particle separations greater than about g = 15 µm the weighted mean frequency is relatively independent of g. However, for g < 15 µm (concentration > 40%) the weighted mean frequency decreases with increasing concentration (a) and decreasing particle spacing (b), which again suggests that closely spaced spheres give rise to a higher proportion of low frequency components when using an omnidirectional detector.

Simulations for a directional detector

In order to simulate the signals arriving at a perfectly directional detector, an initial pressure distribution was simulated using uniformly absorbing spheres with a diameter of 3 µm randomly distributed along a line from (0,-50) to (0,50). A pixel at (0,60) was designated to represent a "line-of-sight" detector. The acoustic propagation of the pressure waves to this detector was simulated as for the omnidirectional detectors using a pseudospectral acoustic model implemented using the k-Wave MATLAB toolbox[20]; as before, the "smooth" function was applied to the initial pressure distribution and the pressure waves were propagated through a three-dimensional non-absorbing homogeneous acoustic medium.

The results of this simulation are shown in Supplementary Figure S5. The first three rows in Supplementary Figure S5 show the results for one, two and three spheres respectively. Here the FFTs of the signals (column c) extend over a broad range up to about 500 MHz and exhibit an envelope similar in shape to the cases shown for the omnidirectional detector in the first three rows of Supplementary Figure S3. This is perhaps unsurprising since in both Supplementary Figure S3 and Supplementary Figure S5 the average separations between the particles are equivalent. However, in the fourth row of Supplementary Figure S5 the nine collinear spheres are spaced by a mean distance equivalent to that for the 80% distribution (fourth row of Supplementary Figure S3) and yet the envelope of the FFT is similar to that for the lower quantities of spheres; in other words for the line-of-sight detector there is no downshifting to lower frequencies as demonstrated in the fourth row of Supplementary Figure S3 for the omnidirectional detector. In fact, for the line-of-sight detector the broad envelope in the FFT is essentially preserved for all cases up to (but not including) the limit of zero particle separation. This is clearly demonstrated in Supplementary Figure S6 where the weighted mean frequencies of the FFTs are plotted for different "concentrations" (numbers N of collinear spheres per 100 μm length) in (a) and for mean particle separations g in (b). With increasing numbers of spheres, the weighted mean frequency does not downshift but rather remains almost constant for large particle separations ($N \leq 7$, $g \geq 10$) since in this regime the frequency spectrum is dominated by the diameter of the particles, which is constant. In the regime where g becomes comparable to the sphere diameter ($7 < N \leq 23$, $10 > g > 1.3$), the particle separation dominates the frequency spectrum so the weighted mean frequency increases with decreasing spacing as might be expected. When N exceeds 23 spheres per 100 μm, the separation g between the spheres is nominally less than 1.3 μm and is effectively zero. Thus, beyond 23 spheres, there is a sudden "jump to continuum" where the particles are no longer individually resolvable and are instead perceived by the detector to be a continuous band. This is reflected by the sudden drop in the weighted mean frequency plotted in Supplementary Figure S6.

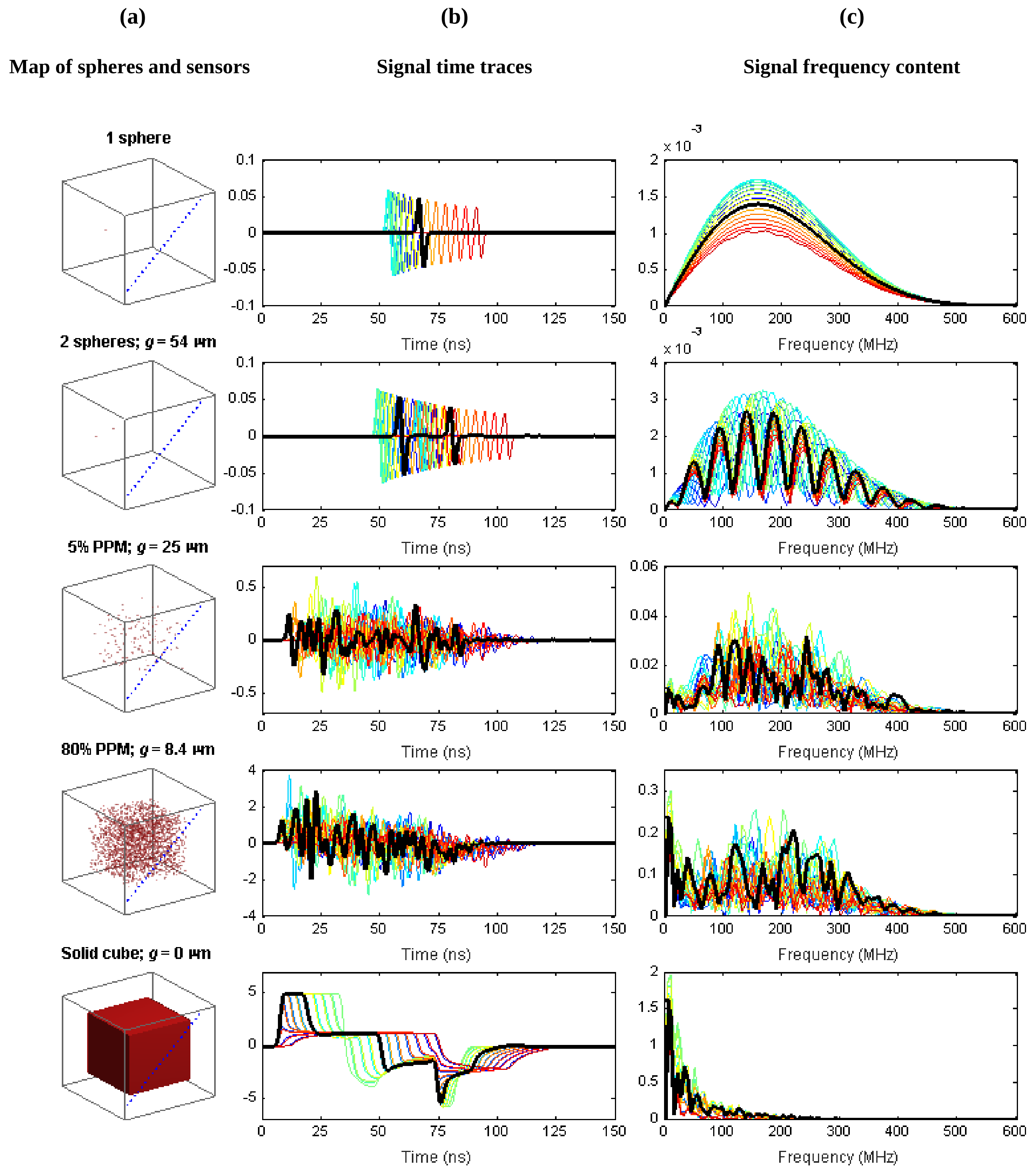

Supplementary Figure S3 Simulations of photoacoustic signals from 3 μm spheres using k-Wave MATLAB toolbox[20]. The "smooth" function was applied to the initial pressure distribution and the pressure waves were propagated through a three-dimensional non-absorbing homogeneous acoustic medium. The 3D plots in column (a) show the distributions of spheres, separated on average by a distance *g* and where relevant expressed as a percentage of the 100% concentration, which is 2.34×10^9 particles/ml (PPM), within a cube of side length 100 μm, which was positioned in the centre of a 150 x 150 x 150 detection grid with a grid point spacing of 1 μm. The solid cube (bottom row) represents the limiting case where all the spheres are touching. A series of 21 detection points were located 15 μm from the edge of the grid along the diagonal of the *x*-*z* plane. Column (b) shows the photoacoustic waveforms recorded at the 21 "sensors" with a time interval of 0.2 ns; the signal from sensor 15 is highlighted as an example. The corresponding fast Fourier transform (FFT) amplitudes are shown in column (c).

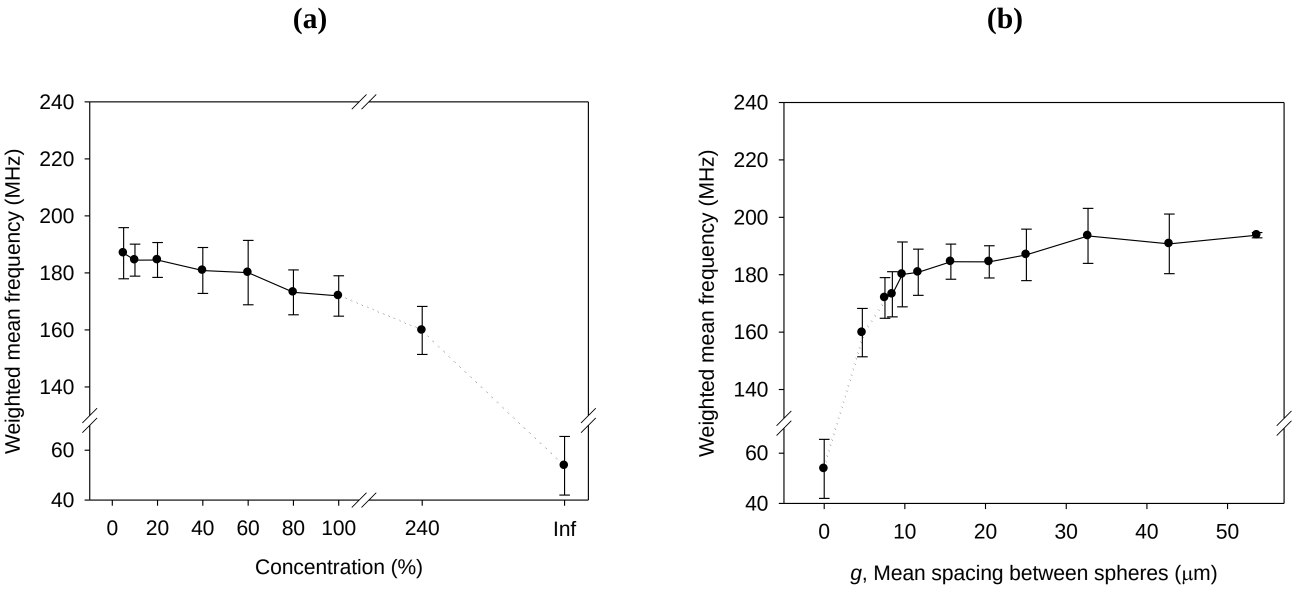

Supplementary Figure S4 Weighted mean frequencies calculated from the fast Fourier transform (FFT) of signals simulated using k-Wave MATLAB toolbox[20] for different concentrations of 3 μm spheres. Examples of the FFTs for the 5% and 80% concentrations are shown in Supplementary Figure S3. For each concentration, weighted mean frequencies were calculated for the FFT of the signals received at each of the 21 simulated sensors. The plots show the mean of the 21 weighted mean values, and the error bars are the standard deviation values. (a) Weighted mean frequencies plotted for simulated random distributions of red spheres ranging from 5% to 100% (equivalent to the experimental concentrations) and also for 240% and infinite (Inf) concentrations. Note that 100% is 2.34 x 10^9 particles/ml (as in the experiments) and that infinite concentration is the case where the spheres have effectively coalesced (g = 0 μm) as represented by the solid cube as shown in the last row of Supplementary Figure S3. (b) Weighted mean frequencies plotted vs. the mean calculated spacing g between the spheres for the same concentrations as in (a) and also for 3%, 1% and 2 spheres (g = 33 μm, 43 μm, and 54 μm respectively).

(a) **(b)** **(c)**

Map of spheres **Signal time traces** **Signal frequency content**

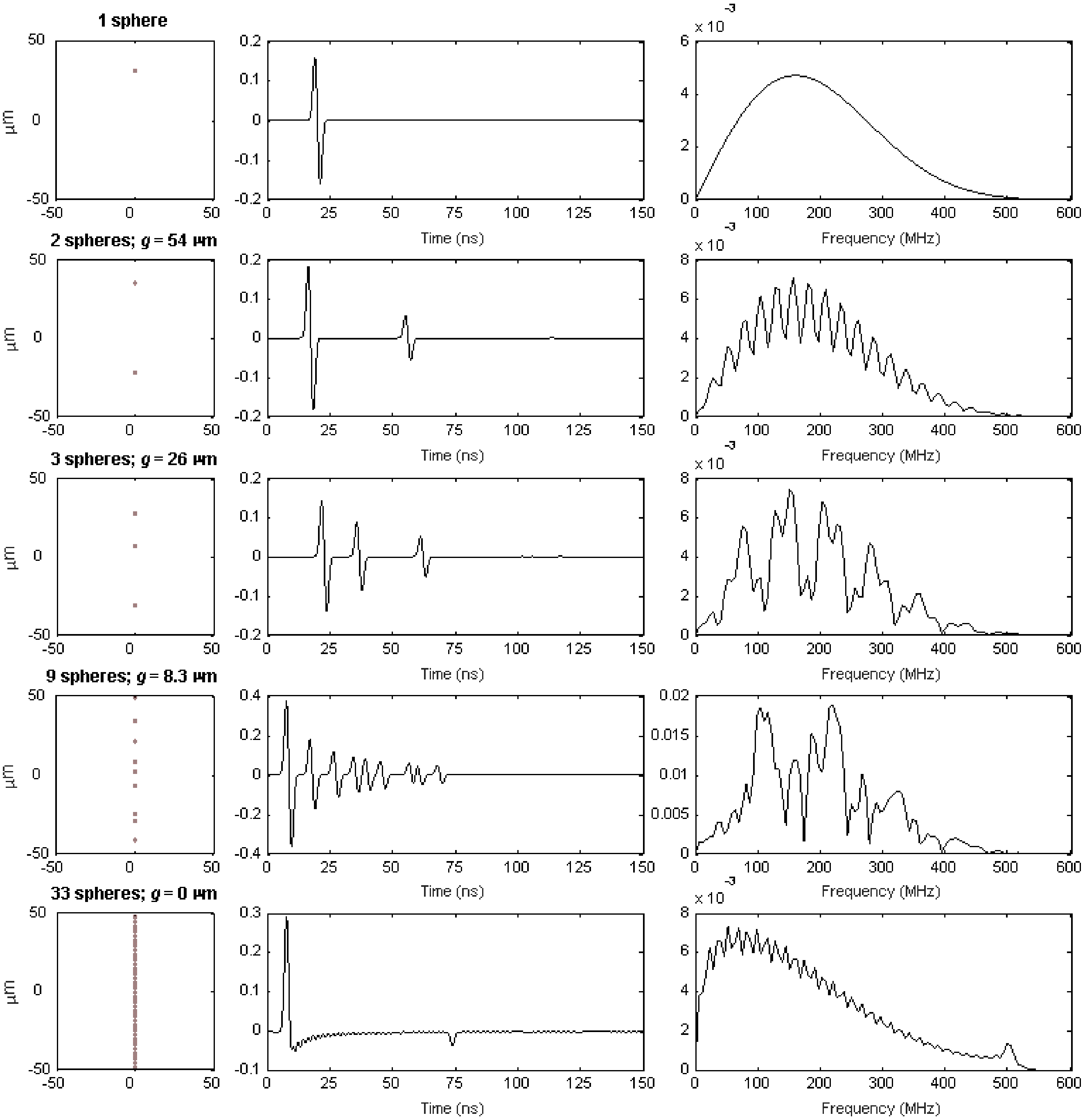

Supplementary Figure S5 Simulations of photoacoustic signals from 3 μm spheres using k-Wave MATLAB toolbox[20]. The “smooth” function was applied to the initial pressure distribution and the pressure waves were propagated through a three-dimensional non-absorbing homogeneous acoustic medium. The images in column (a) show 2D planes through the 3D distributions of spheres placed randomly, separated on average by a distance *g*, along a line of length 100 μm, which was positioned in the centre of a 150 x 150 x 150 detection grid with a grid point spacing of 1 μm. Column (b) shows the photoacoustic waveforms recorded at a detector placed at (x,y) = (0,60). The corresponding fast Fourier transform (FFT) amplitudes are shown in column (c).

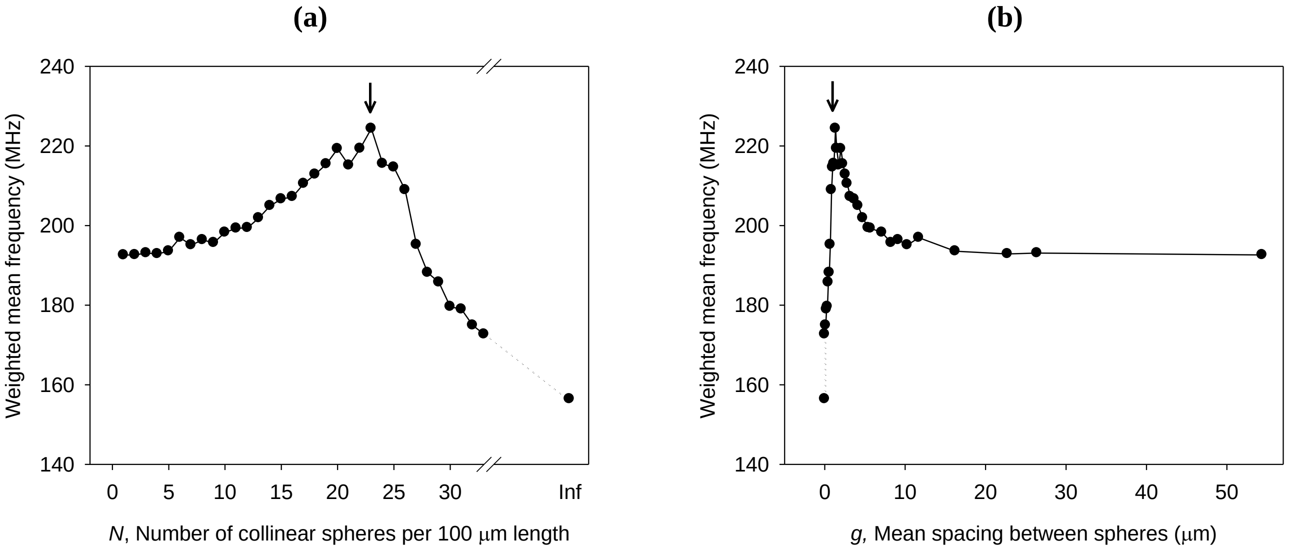

Supplementary Figure S6 (a) Weighted mean frequencies calculated from the fast Fourier transform (FFT) of signals simulated using k-Wave MATLAB toolbox[20] for different quantities *N* of 3 μm collinear spheres per 100 μm. The infinite case (Inf) the individual spheres have formed a continuum which is simulated by a solid smoothed cuboid with a cross-sectional area equivalent to that of a single sphere but with a length extending over the 100 μm domain. Examples of the FFTs for 1, 2, 3, 9 and 33 spheres are shown in Supplementary Figure S5. (b) Weighted mean frequencies for the same simulations as in (a) but plotted vs. the mean spacing *g* between the spheres (excluding the 1-sphere case). The arrows in (a) and (b) mark the point at which the spheres coalesce to give a mean particle separation that is effectively zero. This occurs at a value of *g* slightly greater than zero, since *g* is calculated using the nominal diameter of 3 μm rather than the slightly larger sphere diameter that is produced as a consequence of the smoothing function applied to the initial pressure source distribution (k-Wave "smooth" function, Blackman window).
